# Neurobiological mechanisms of the effect of exercise on depressive disorder: analysis using CiteSpace

**DOI:** 10.3389/fpsyt.2025.1600286

**Published:** 2025-09-02

**Authors:** Haiying Yang, Jiayi Xu, Lei Hou, Dejian Huang

**Affiliations:** School of Physical Education, Southwest Jiaotong University, Chengdu, Sichuan, China

**Keywords:** exercise, depressive disorder, neurobiological mechanisms, BDNF (brain-derived neurotrophic factor), scientometric analysis

## Abstract

Research on the neural mechanisms of exercise interventions for depressive disorder has evolved significantly over the past decade; however, there remains a lack of scientometric synthesis tracking these changes, including analyses of researcher networks and scientific productivity up to December 31, 2024. We searched the Web of Science Core Collection using specific search terms and conducted a comprehensive scientometric analysis to systematically examine the evolutionary pathways, collaborative networks, and core mechanisms in the relevant literature from 2006 to 2024. Our analysis included 170 core studies, and the co-cited reference network identified seven clusters with well-structured networks (Q=0.9299) and highly confident clustering (S=0.9794). Results revealed that exercise exerts antidepressant effects through modulation of neurotransmitter systems (e.g., 5-HT, GABA receptor subtypes), up-regulation of brain-derived neurotrophic factor (BDNF) expression, and enhancement of hippocampal neuroplasticity. The research development was divided into three distinct phases: the early phase (2006-2014), which validated the effects of exercise on monoamine transmitters; the middle phase (2015–2019), which confirmed that aerobic exercise can produce effects comparable to pharmaceutical interventions; and the recent phase (post-2020), which has focused on gene-environment interactions and metabolism-neuraxis mechanisms. The collaborative network has expanded from the initial Swiss-German axis to a global level. Future research should integrate metabolomics and neuroimaging technologies to develop precise exercise prescriptions and optimize real-time intervention feedback using digital tools, thereby providing theoretical frameworks and translational pathways to enhance non-pharmaceutical intervention strategies.

## 1 Introduction

Depressive disorder, a debilitating psychiatric condition affecting over 185 million individuals globally, is characterized by dysregulated neurobiological pathways, including impaired monoaminergic neurotransmission, diminished neurotrophic signaling, and structural alterations in limbic circuits (1). Exercise has emerged as a pivotal non-pharmacological intervention, with meta-analyses demonstrating its efficacy in alleviating depressive symptoms through mechanisms such as serotonin (5-HT) and gamma-aminobutyric acid (GABA) modulation, hippocampal neurogenesis, and brain-derived neurotrophic factor (BDNF) upregulation (2–4). Early research efforts focused on validating exercise-induced monoaminergic adaptations, while subsequent studies expanded to comparative efficacy trials against pharmacological treatments, such as selective serotonin reuptake inhibitors (SSRIs) (5, 6).

In recent years, the application of “omics” approaches—such as metabolomics, proteomics, genomics, and neuroimaging—has become increasingly essential for elucidating the molecular and systemic effects of exercise on the brain. These integrative methods provide a deeper understanding of the neurobiological mechanisms underlying exercise’s antidepressant effects, which have remained elusive in traditional psychological or clinical research. While metabolomics and other omics approaches were not directly analyzed in this study, their integration into the field of exercise research has profound implications. For example, metabolomics has identified biomarkers such as lactate and ketone bodies, which modulate neuroplasticity and influence mood regulation pathways (7). In our scientometric analysis, these methodologies are integral to understanding how molecular changes induced by different exercise modalities could inform tailored, genotype-guided interventions in the future.

Moreover, integrating neuroimaging with omics data has enhanced our understanding of exercise-induced changes in brain structure and function. Studies show that regular physical activity boosts BDNF levels and hippocampal volume—both of which are strongly associated with depression (8). This combined approach links molecular shifts to neuroanatomical adaptations, advancing our knowledge of exercise as a potential treatment for depression. Importantly, the application of omics techniques in exercise-depression research aligns with the growing trend of personalized medicine. By allowing for individualized analysis of molecular responses to exercise, omics research paves the way for the development of optimized, tailored therapeutic strategies (7, 8).

The exponential growth of interdisciplinary evidence on exercise-induced neurobiological adaptations in depression necessitates novel approaches to synthesize and visualize trends within knowledge domains (9). Nakagawa et al. (2019) recently proposed a framework termed research weaving, which integrates systematic mapping with bibliometric analysis to simultaneously evaluate evidence synthesis and academic influence (10). This framework combines systematic mapping and bibliometric analysis to inform the development of the field, the influence of research papers and their interconnections, and to visualize content across and within publications. Systematic mapping allows one to visualize and map the evolution of research over time, while bibliometric analyses measure how evidence is interconnected and the influence of authors via two complementary methods: performance analysis (e.g., citation counts, h-index) and bibliometric mapping (e.g., co-citation networks, keyword co-occurrence) (11). This hybrid approach, defined as scientometric analysis, provides a comprehensive synthesis of a research field’s trajectory, identifies redundancies and gaps, and highlights potential moderators such as methodological heterogeneity, publication bias, or limitations in evidence sources (12).

Unlike traditional narrative or systematic reviews, which are constrained by manual selection and subjective interpretation of limited datasets, scientometric analysis leverages large-scale bibliographic metadata to map evolving research structures, influential knowledge domains, and emerging conceptual trends (12, 13). While it does not reveal new biological mechanisms per se, this method identifies how such mechanisms have been prioritized, contextualized, and integrated across disciplines over time. For example, co-citation and keyword co-occurrence networks can expose hidden linkages between previously disconnected research topics—such as the convergence of BDNF signaling with gut-brain axis research—thereby highlighting conceptual turning points and underexplored areas with high potential for innovation (9). In this way, scientometric mapping not only facilitates comprehensive field synthesis but also enhances strategic planning for future interdisciplinary investigations.

Within the context of exercise and depressive disorder, scientometric analysis is particularly valuable given the rapid evolution of neurobiological mechanisms (e.g., BDNF signaling, hippocampal neuroplasticity) and the growing urgency to translate mechanistic discoveries into personalized interventions (14). The integration of metabolomics, neuroimaging, and digital health tools further underscores the need for a data-driven framework to optimize precision exercise prescriptions and real-time intervention feedback (15, 16). While this study does not directly analyze omics datasets, the scientometric methodology allows us to trace the emergence and influence of these tools over time by examining their frequency in co-citation networks and keyword clusters (17). Omics approaches are pivotal for elucidating how exercise influences metabolic, inflammatory, and neuroplastic pathways at a systems level, providing mechanistic depth to the behavioral outcomes observed in clinical studies (18, 19). As such, their growing relevance within the research network justifies their inclusion as emerging conceptual frontiers with high translational potential (20). Although bibliometric studies on exercise and mental health have been conducted within specific regions (e.g., European clinical trials, North American cohort studies) (21), no global scientometric analysis has comprehensively mapped the structural and temporal dynamics of exercise-induced neurobiological mechanisms in depressive disorder.

Given the findings discussed above, we employed CiteSpace to construct cross-temporal and cross-disciplinary knowledge maps, systematically analyzing literature on the neurobiological mechanisms underlying exercise interventions for depressive disorder from 2006 to 2024. By leveraging CiteSpace, we not only generated dynamic visualizations of co-citation networks, keyword co-occurrence, and collaborative networks but also captured the evolution of research hotspots—from early studies focusing on single neurotransmitter mechanisms to more recent investigations into BDNF regulation, gene–environment interactions, and digital health integration. This novel mapping approach reveals the progressive diversification and complexity of exercise’s neurobiological impact on depression, thereby offering a clear theoretical basis for the development of personalized exercise prescriptions and real-time intervention strategies. Such insights address existing gaps between pharmacological and non-pharmacological treatments for depression and pave the way for more precise clinical applications.

## 2 Methodology

### 2.1 Objectives

Our primary objective was to systematically map how research on the neural mechanisms of physical activity interventions for depressive disorder has evolved over time and to identify the evolution of key research themes through the use of a network of co-cited references and a network of co-occurring keywords, and secondary objectives were to provide clinicians and researchers with metrics of the research network (countries, organisations, authors, and journals) and to detect research richness, gaps, emerging trends, biases, and limitations.

### 2.2 Search strategy and data analysis

We searched the Web of Science Core Collection (WOSCC) as the most comprehensive database for scientometric analyses (22).

A comprehensive search strategy was employed using both MeSH (Exercise, Depressive Disorders, and Neurobiology Subject Headings) terms and keywords. The MeSH terms were selected based on their relevance to the research topic, ensuring a broad coverage of relevant literature. The full search terms were provided in the study protocol ((TS=(Exercises) OR TS=(Exercise, Physical) OR TS=(Exercises, Physical) OR TS=(Physical Exercise) OR TS=(Physical Exercise) OR TS=(Physical Exercises) OR TS=(Physical Exercises) OR TS=(Physical Activity) OR TS=(Physical Activity) OR TS=(Physical Activity) OR TS=(Physical Activity) OR TS= (Physical Activity) OR TS=(Activities, Physical) OR TS=(Activity, Physical) OR TS=(Physical Activities) OR TS=(Physical Activities) OR TS=(Exercise, Aerobic) OR TS=(Aerobic Exercise) OR TS=(Aerobic Exercises) OR TS=(Exercises, Aerobic) OR TS=(Exercise, Isometric) OR TS=(Exercises, Isometric) OR TS=(Isometric) Exercises) OR TS=(Isometric Exercise) OR TS=(Acute Exercise) OR TS=(Acute Exercises) OR TS=(Exercise, Acute) OR TS=(Exercises, Acute) OR TS=(Exercises, Acute) OR TS=(Exercise Training) OR TS=(Exercise Trainings)) And ((TS=(Depressive Disorders) OR TS=(Disorder, Depressive) OR TS=(Disorders, Depressive) OR TS=(Disorders, Depressive) OR TS=(Neurosis, Depressive) OR TS=(Depressive Neuroses) OR TS=(Depressive Neurosis) OR TS=(Neuroses, Depressive) OR TS=(Depression, Endogenous) OR TS=(Depressions, Endogenous) OR TS=(Endogenous Depression) OR TS=(Endogenous Depressions) OR TS=(Melancholia) OR TS=(Melancholias) OR TS=(Unipolar Depression) OR TS=(Depression, Unipolar) OR TS=(Depressions, Unipolar) OR TS=(Unipolar Depressions) OR TS=(Depressive Syndrome) OR TS=(Depressive Syndromes) OR TS=(Syndrome, Depressive) OR TS=(Syndromes, Depressive) OR TS=(Depression, Neurotic) OR TS=(Depressions, Neurotic) OR TS=(Depressions, Neurotic) OR TS=(Neurotic Depression) OR TS=(Neurotic Depressions)) AND ((TS=(neurobiology) OR TS=(brain function) OR TS=(neurophysiology) OR TS=(neuroscience) OR TS=(biological mechanisms) OR TS=(neurotransmitters) OR TS=(brain plasticity) OR TS=(brain-derived neurotrophic factor) OR TS=(amygdala) OR TS = (prefrontal cortex)). The source of the database was restricted to WOS, the publication type was ‘article’, the language type was English, and WOSCC extracted complete records with cited references published up to 31 December 2024 and converted them into tab-delimited plain text files. Duplicates were eliminated using CiteSpace.

**Figure 1:**
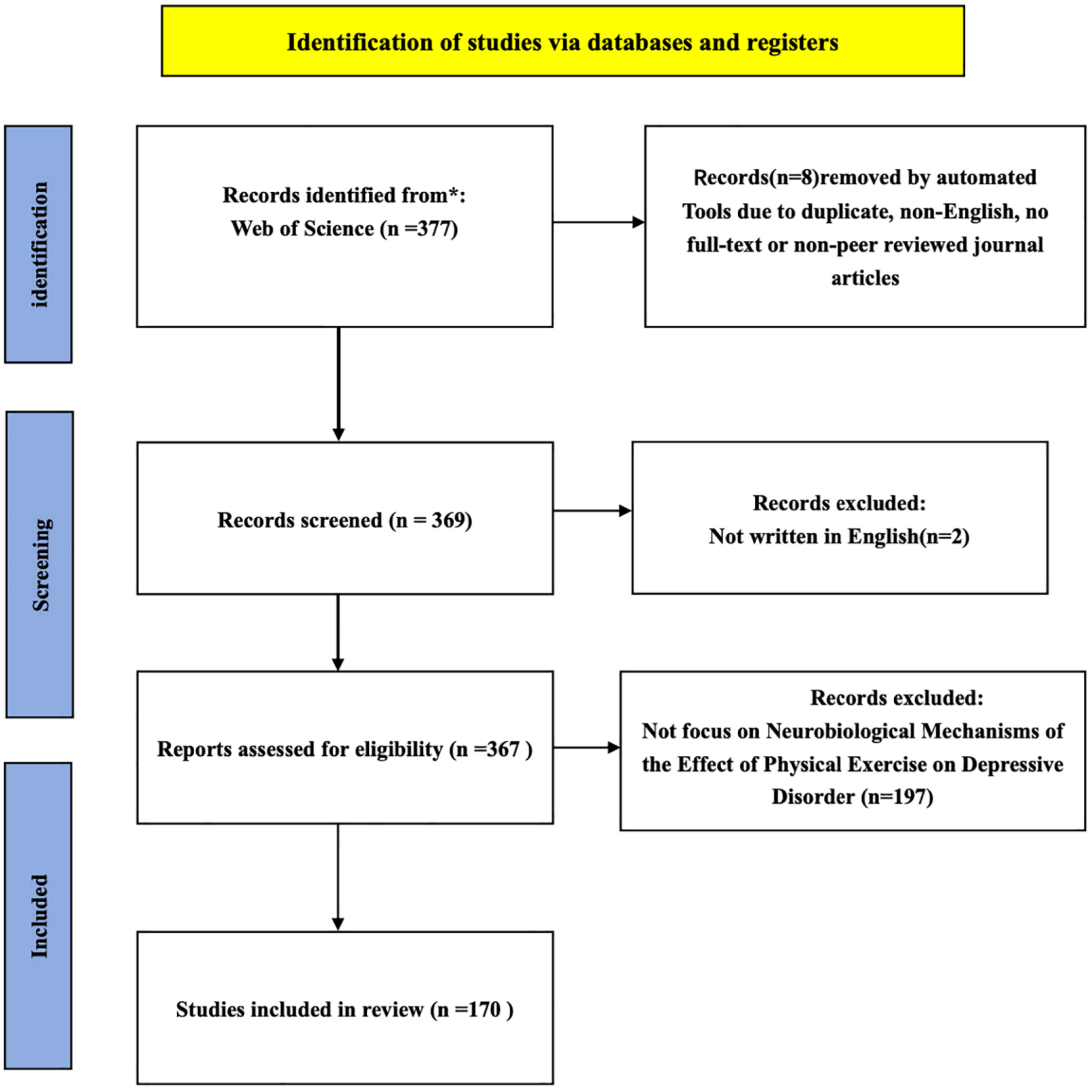
PRISMA flow diagram.

Once the search was conducted, the extraction and the reasons reported for excluding articles were processed in the flowchart (**Figure 1**). The screening process was conducted by two independent reviewers. First, titles and abstracts of the identified articles were assessed to determine relevance. After an initial screening, 2 non-English articles were excluded, leaving 367 articles for further assessment. During the second phase, the full texts of these articles were reviewed, and 197 studies were excluded because they were not relevant to the topic of neural mechanisms of physical activity interventions in depression. Ultimately, 170 studies were eligible and included in the review. Discrepancies between reviewers were resolved through discussion and consensus.

### 2.3 Data analysis

The study used the bibliometric software CiteSpace (version 6.4.R1) (9) to perform a thorough analysis of scientific literature. The analysis examined various aspects, such as author contributions, journal impact, reference networks, international collaborations, institutional affiliations, and patterns of keyword co-occurrence. To create the scientific knowledge graph, the network topology was divided into two main types based on relationship structures: directed graphs, which include direct citation networks that show asymmetric citation relationships among documents, and undirected graphs, which consist of co-citation and co-occurrence networks that illustrate symmetric relationships between elements. A systematic comparative analysis by Boyack and Klavans (2010) emphasized the differences between direct citation and co-citation networks in how they represent knowledge structures (23).

The bibliometric results included citation counts, co-citation, and co-occurrence metrics. Co-citation count refers to how often two published works are cited together in later articles (24). In this study, three bibliometric indicators were used: citation frequency, which reflects the influence of literature; co-citation intensity, which indicates the strength of knowledge connections; and co-occurrence frequency, which shows the degree of conceptual relationship. The co-citation network has a unique advantage in systematic reviews due to its dynamic nature, which effectively tracks the evolution of knowledge communities (25). At the same time, the co-occurrence network clarifies relationships among variables using graph-theoretic methods.

The CiteSpace platform offers various dimensional metrics to effectively evaluate network characteristics. It initially employed temporal indicators, particularly Citation Burst, to track the chronological development of research focuses. Following this, structural indicators were used to evaluate how well nodes connect within the network. Nodes with high centrality often serve as vital knowledge hubs that link different clusters. Additionally, composite indicators such as Modularity Q and Silhouette S metrics (26) were integrated to assess the overall structure of the network. The Q-value, which ranges from 0 to 1, indicates network modularity, with values above 0.3 suggesting a significant clustering structure. In contrast, the S-value, which ranges from -1 to 1, measures the homogeneity of clusters; thresholds of 0.3, 0.5, and 0.7 reflect increasing confidence in the clustering. An S-value of 1 may indicate that a cluster is isolated, necessitating careful interpretation within the specific domain context.

Bibliometric techniques were utilized to analyze scientific literature from various perspectives. For cluster labeling, a system was created by extracting noun phrases from the keyword lists of each literature cluster, based on significance determined by likelihood ratio tests (p < 0.001). Data collection and analysis adhered to standardized protocols: CiteSpace version 6.4.R1 was employed for multidimensional knowledge graph analysis, concentrating on international collaboration networks at both country and institutional levels, co-citation networks that highlight author and literature clustering, and author keyword co-occurrence networks. Furthermore, journal impact factor data were sourced from the 2023 Journal Citation Reports (JCR) of the Web of Science, in terms of index calculation, we adopt the g-index (g-index) as the core index for author impact assessment, which, by integrating the citation distribution characteristics (27), effectively corrects the over-sensitivity of the h-index to highly cited papers, and ensures the weight of high-impact papers while taking into account the low/zero-citation literature’s CiteSpace’s time slicing function removes empty time intervals through an automatic optimisation algorithm to achieve intelligent compression of the analysis time domain.

## 3 Results

### 3.1 Analysis of co-cited references: research and most cited paper clusters

#### 3.1.1 Research clusters

**Figure 2:**
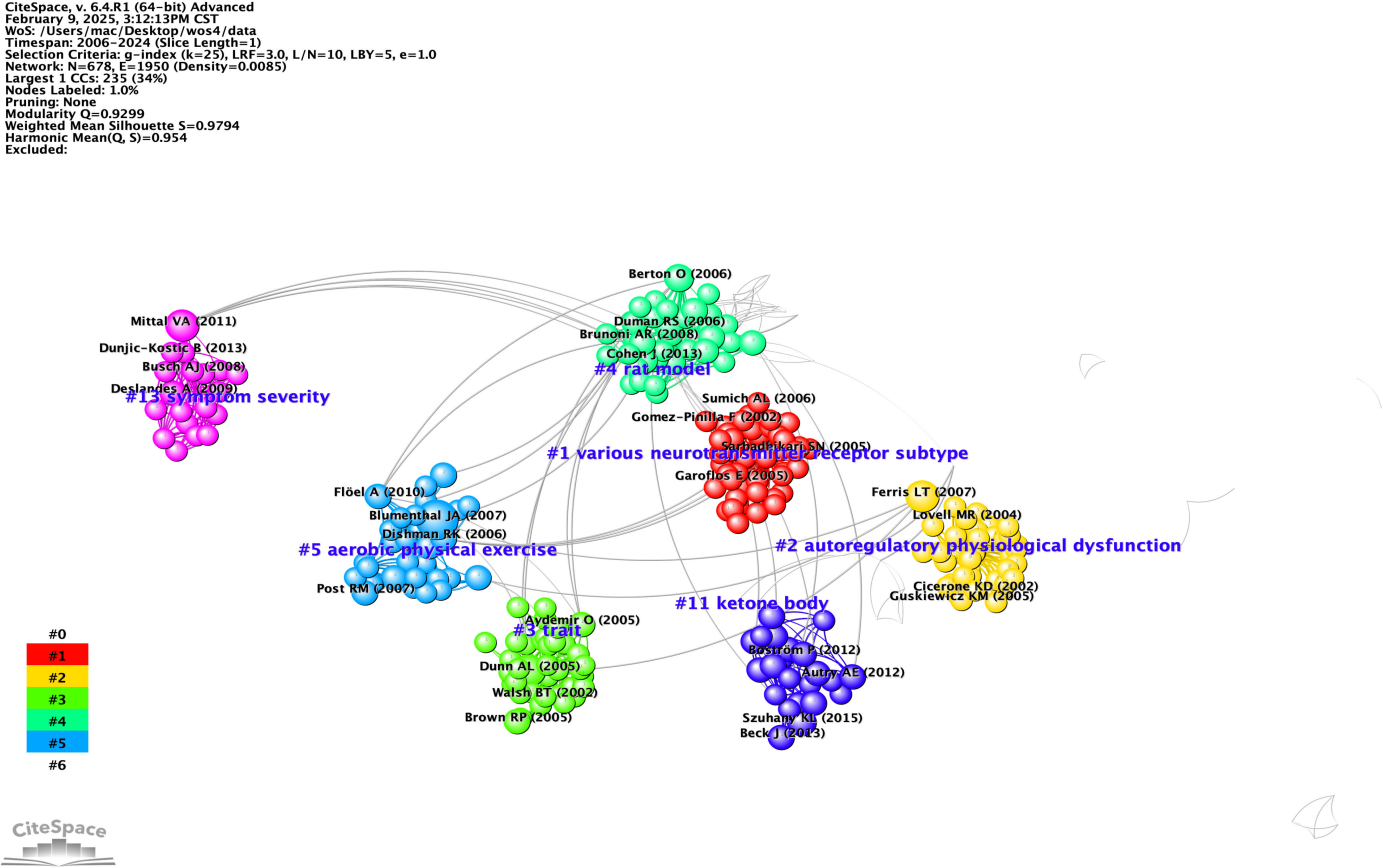
Co-citation references network (2006-2024) and correspondent clustering analysis obtained with CiteSpace. Co-citation reference network with cluster visualization and burstness of hotspots. The size of a node (article) is proportional to the number of times the article has been co-cited.

After executing Citespace, we obtained the visualization depicted in **Figure 2**. By selecting ‘T’ as the source for labeling and employing the log-likelihood ratio as the clustering method, seven distinct clusters emerged. **Figure 2** reveals a modularity Q-value of 0.9299, with an average profile S-value of 0.9794. Notably, all seven principal clusters display ‘Side Image’ values nearing 1, signifying a high caliber of clustering within this knowledge graph. Comprehensive details regarding the clusters are presented in **Figure 2** and **Table 1**, starting with Neurotransmitter Receptor Subtypes (Cluster #1), which investigates the influence of various neurotransmitter receptor subtypes in the modulation of stress and exercise, as noted by Sarbadhikari (28). Initial studies conducted between 2005 and 2006 concentrated on the impacts of neurotransmitter systems, such as 5-HT and GABA receptors, on mood and cognition. These studies suggested that exercise enhances cerebral function by adjusting receptor activity, thereby establishing a theoretical framework for further investigations into the molecular mechanisms underpinning exercise interventions, particularly in relation to Cluster #5, which focuses on Aerobic Exercise.

**Table 1:** Details of knowledge clusters.

ID	Size	Silhouette	Label (LSI)	Label (LLR)	Label (MI)	Average Year
1	48	1	moderate exercise and chronic stress produce counteractive effects on different areas of the brain by acting through various neurotransmitter receptor subtypes: a hypothesis	various neurotransmitter receptor subtype (5.82, 0.05)	various neurotransmitter receptor subtype (0.09)	2004
2	41	1	regulatory and autoregulatory physiological dysfunction as a primary characteristic of post concussion syndrome: implications for treatment	autoregulatory physiological dysfunction (7.75, 0.01)	autoregulatory physiological dysfunction (0.04)	2004
3	38	0.985	effect	trait (7.34, 0.01)	acute response (0.37)	2004
4	37	0.958	rat model	rat model (9.07, 0.005)	structured exercise (0.97)	2009
5	30	0.935	neurocognitive dysfunction	aerobic physical exercise (12.21, 0.001)	bdnf genotype moderate (0.29)	2008
11	22	0.964	neurotrophic factor	ketone body (13.04, 0.001)	various neurotransmitter receptor subtype (0.23)	2013
13	19	1	result	symptom severity (7.78, 0.01)	acute exercise (0.32)	2009

Self-regulatory Physiological Dysfunctions (Cluster #2) underscore the significance of physiological self-regulation, such as autonomic nervous system function, in neurorehabilitation, advocating for the implementation of non-pharmacological therapies like exercise therapy in trauma recovery, as highlighted by Leddy (29). Trait and Mood Regulation (Cluster #3) examines the influence of individual traits, such as the BDNF genotype, on the antidepressant effectiveness of exercise, transitioning from a one-size-fits-all approach to personalized medicine, also noted by Leddy in 2007. This shift occurred between 2007 and 2009, emphasizing gene-environment interactions, such as yoga efficacy for specific depressive subtypes (30).

Cluster #4, which is based on rat models, employs animal studies to validate the effects of exercise on brain-derived neurotrophic factor (BDNF) levels and depressive behaviors. This cluster provides foundational research insights from 2006 to 2015 that inform the design of clinical trials, as illustrated in Cluster #5, signalling the rise of translational medicine (31–34). The latter cluster, focusing on Aerobic Exercise Interventions, presents clinical studies conducted between 2007 and 2012 that demonstrate aerobic exercise’s efficacy comparable to that of medication for treating depression and bipolar disorder. This evidence advocates for the inclusion of aerobic exercise in treatment guidelines and examines biomarkers, such as BDNF, as potential indicators of treatment effectiveness (35–37). 

Meanwhile, Cluster #11 delves into the emerging field of ketone bodies, particularly β-hydroxybutyric acid, and their role in mediating exercise-induced BDNF expression and neuroprotection. This cluster highlights the significance of metabolites in psychiatric disorders, bridging exercise science with metabolomics, and paving the way for innovative combined nutrition and exercise interventions (38, 39).

Cluster #13, which focuses on Symptom Severity Assessment, explores both the immediate and long-term impacts of exercise on the severity of depressive symptoms and cognitive functioning. Research conducted within this cluster from 2011 to 2013 has increasingly emphasized the quantification of efficacy, integrating behavioral methods like dual-task paradigms to enhance exercise prescriptions aimed at specific symptoms, such as cognitive impairment associated with geriatric depression (40, 41).

Overall, three significant trends emerge from this body of research: the first trend reflects a shift from broad neurotransmitter studies (#1) to a more detailed examination of molecular mechanisms, including brain-derived neurotrophic factor (BDNF) and ketone bodies (#4, #11), ultimately leading to personalized medicine approaches (#3, #5). The second trend highlights the movement towards precision interventions, concentrating on genotypes (#3), biomarkers (#5), and symptom classification (#13) to refine treatment strategies. Lastly, the third trend points to the metabolism of ketone bodies (#11) as a potential breakthrough in understanding the mechanisms behind exercise as an antidepressant.

This analysis identifies the key themes present in each cluster. Cluster #1 highlights the importance of neurotransmitter receptors, such as serotonin and GABA, in managing stress responses and enhancing mood, which sets the stage for molecular research that corresponds with Cluster #5’s investigation into the neurochemical impacts of exercise. Cluster #2 underscores the vital function of the autonomic nervous system in neurorehabilitation, promoting non-drug therapies like exercise to aid trauma recovery and merging exercise science with psychological rehabilitation. Cluster #3 examines how personal characteristics, including BDNF genotype, influence the effectiveness of exercise as an antidepressant, indicating a move towards more tailored treatment approaches.

In Cluster #4, animal studies reveal that exercise boosts BDNF levels and neuroplasticity, thereby linking fundamental science to clinical trial design and confirming that aerobic exercise can yield results comparable to those of pharmacological treatments. Cluster #11 examines how ketone bodies, particularly β-hydroxybutyric acid, enhance BDNF expression and neuroprotection, implying that combining nutritional strategies with exercise could effectively tackle psychiatric disorders. Cluster #13 assesses both the short-term and long-term impacts of exercise on depressive symptoms and cognitive function, suggesting that dual-task paradigms be used to customize interventions for specific symptoms. Collectively, these findings reflect a transition from general neurotransmitter mechanisms to specific molecular processes and personalized treatment strategies, paving the way for exciting new interdisciplinary research opportunities in psychiatric care.

#### 3.1.2 Most co-cited papers

**Figure 3:**
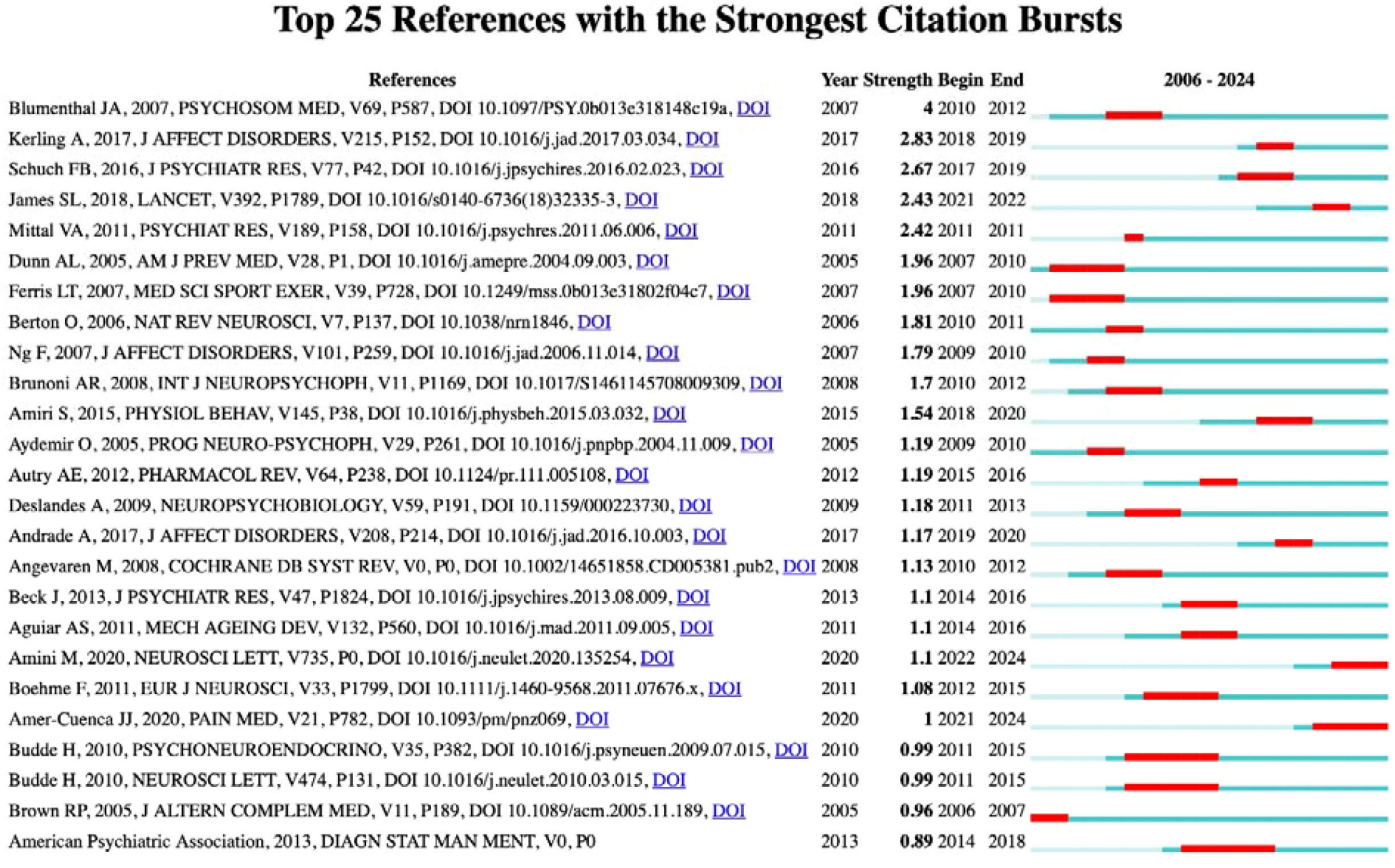
Top 25 Reference with the strongest citation bursts.

Citation burst analysis provides insights into the temporal dynamics of research influence. **Figure 3** presents the top 25 references with the strongest citation bursts from 2006 to 2024, revealing distinct periods of heightened research activity. The analysis shows notable citation bursts occurring during 2010-2012 and a particularly active period from 2017-2019, indicating evolving research priorities and methodological approaches in the field over time.

**Table 2** highlights the ten most co-cited references in the field, with Schuch et al. (2016) leading the list, having been cited 9 times in the reference list and accumulating a total of 98 citations for its systematic evaluation of exercise as a treatment for depression (42). Following closely, Blumenthal et al. (2007) conducted a comparison between exercise and medication (43), while Kerling et al. (2017) proposed a neurotrophic hypothesis, suggesting that specific types of exercise may enhance BDNF levels and promote neuronal growth (44). Foundational studies such as those by Duman et al. (2006), which has garnered 2,976 citations (45), and the Cochrane review by Cooney et al. (2013) with 863 citations, provide robust support for exercise interventions (46).

**Table 2:** The top 10 most cited reference.

Number of citations in the network	Number of citations in the literature	Cited reference	Year	Source	Vol	Page	Title	Doi	Type of paper	Related cluster in Figure 1
9	98	Schuch FB	2016	Journal of Psychiatric Research	77	42	Exercise as a treatment for depression: A meta-analysis adjusting for publication bias	10.1016/j.jpsychires.2016.02.023	Meta-analysis	9
7	98	Blumenthal JA	2007	Psychosomatic Medicine	69	587	Exercise and Pharmacotherapy in the Treatment of Major Depressive Disorder	10.1097/PSY.0b013e318148c19a	Clinical Trial	7
5	57	Kerling A	2017	Journal of Affective Disorders	215	152	Exercise increases serum brain-derived neurotrophic factor in patients with major depressive disorder	10.1016/j.jad.2017.03.034	Clinical Trial	5
4	74	Dunn AL	2005	American Journal of Preventive Medicine	28	1	Exercise treatment for depression - Efficacy and dose response	10.1016/j.amepre.2004.09.003	Clinical Trial	4
4	159	American Psychiatric Association	2011	Journal of Psychiatric Research	189	158	Diagnostic and Statistical Manual of Mental Disorders	10.1016/j.psychres.2011.06.006	Reference Book	4
4	98	James SL	2018	The Lancet	392	1789	Global, regional, and national disability-adjusted life-years (DALYs) for 359 diseases and injuries and healthy life expectancy (HALE) for 195 countries and territories, 1990–2017: a systematic analysis for the Global Burden of Disease Study 2017	10.1016/s0140-6736 (18)32335-3	Systematic Analysis and Review	4
4	74	Ferris LT	2007	Medicine and Science in Sports and Exercise	39	728	The effect of acute exercise on serum brain-derived neurotrophic factor levels and cognitive function	10.1249/mss.0b013e31802f04c7	Clinical Trial	4
4	863	Cooney GM	2013	Cochrane Database of Systematic Reviews	0	0	Exercise for depression	10.1002/14651858.CD004366.pub6	Review	4
3	394	Berton O	2006	Nature Reviews Neuroscience	7	137	New approaches to antidepressant drug discovery: beyond monoamines	10.1038/nrn1846	Review	3
3	2976	Duman	2006	Biological Psychiatry	59	1116	A neurotrophic model for stress-related mood disorders	10.1016/j.biopsych.2006.02.013	Review	3

Additionally, Berton et al. (2006) contributed to the discourse with 394 citations, focusing on non-monoamine targets for antidepressant development (47). A burst analysis indicated that the highest citation intensities were for Blumenthal et al. (4.0), Kerling et al. (2.83), and Schuch et al. (2.67). Notably, early hotspots in research emerged from studies comparing exercise and medication between 2010 and 2012, while a surge in meta-analyses from 2017 to 2019 significantly propelled research in this area. The core themes identified include the treatment of depression through exercise interventions, the exploration of molecular mechanisms such as the neurotrophic hypothesis and non-monoamine targets, and the introduction of innovative technological approaches, as illustrated by Amini et al. (48). Overall, the literature from the years 2007, 2016, 2017, and 2020 reflects a high burst intensity, underscoring substantial contributions to the advancement of exercise interventions for depression and the clarification of their Underlying mechanisms.

### 3.2 Keyword co-occurrence analysis

**Figure 4:**
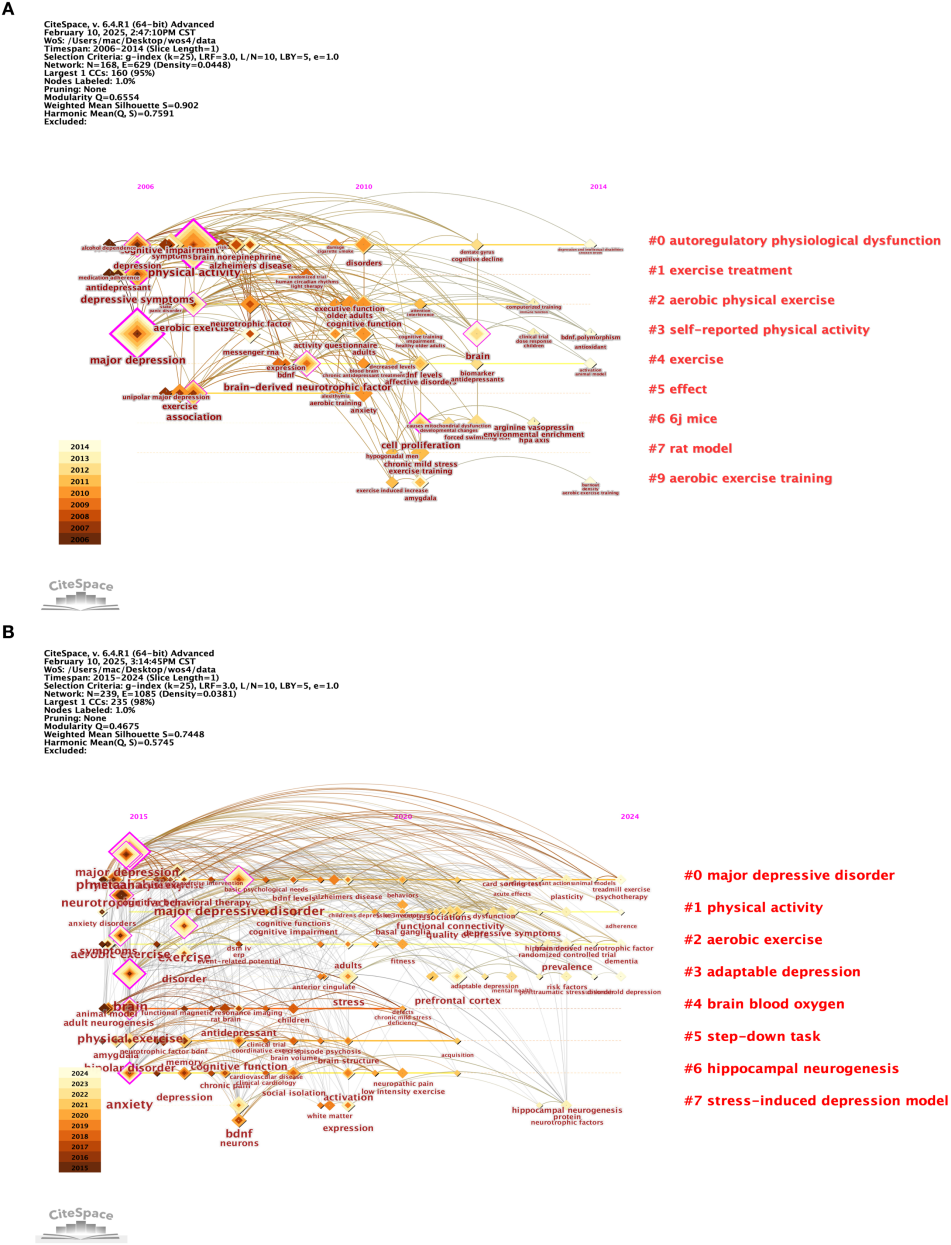
**(A, B)** Timeline visualisation of co-occurring author keyword networks [**(A)** 2006-2014 and **(B)** 2015-2024]. Nodes denote keywords and colours show the average year of publication for each node,the size of the nodes is proportional to the burstiness of the keyword co-occurrence,the co-occurrence network is weighted according to the total link strength of the different keyword nodes and scored according to the average year of publication. Cluster titles are marked in red on the far right of the timeline graph.

To investigate research focal points and trends, we conducted an analysis of the most frequently cited keywords by generating a co-occurring keywords network diagram using CiteSpace, as illustrated in **Figure 4A**. This analysis revealed nine keyword clusters, with the most prominent ones being “major depression,” “aerobic exercise,” and “brain-derived neurotrophic factor” during the period from 2006 to 2014, referred to as cluster A. In the following period from 2015 to 2024, represented as cluster B, a similar network diagram shown in **Figure 4B** displayed eight clusters, which included “Major Depressive Disorder,” “Physical Activity,” “Aerobic Exercise,” “Adaptable Depression,” “Step-down Task,” “Hippocampal Neurogenesis,” and “Stress-induced Depression Model.” Both keyword networks from the two periods demonstrated significant silhouette scores greater than 0.6 and satisfactory modularity scores, indicating a robust structure.

**Figure 5:**
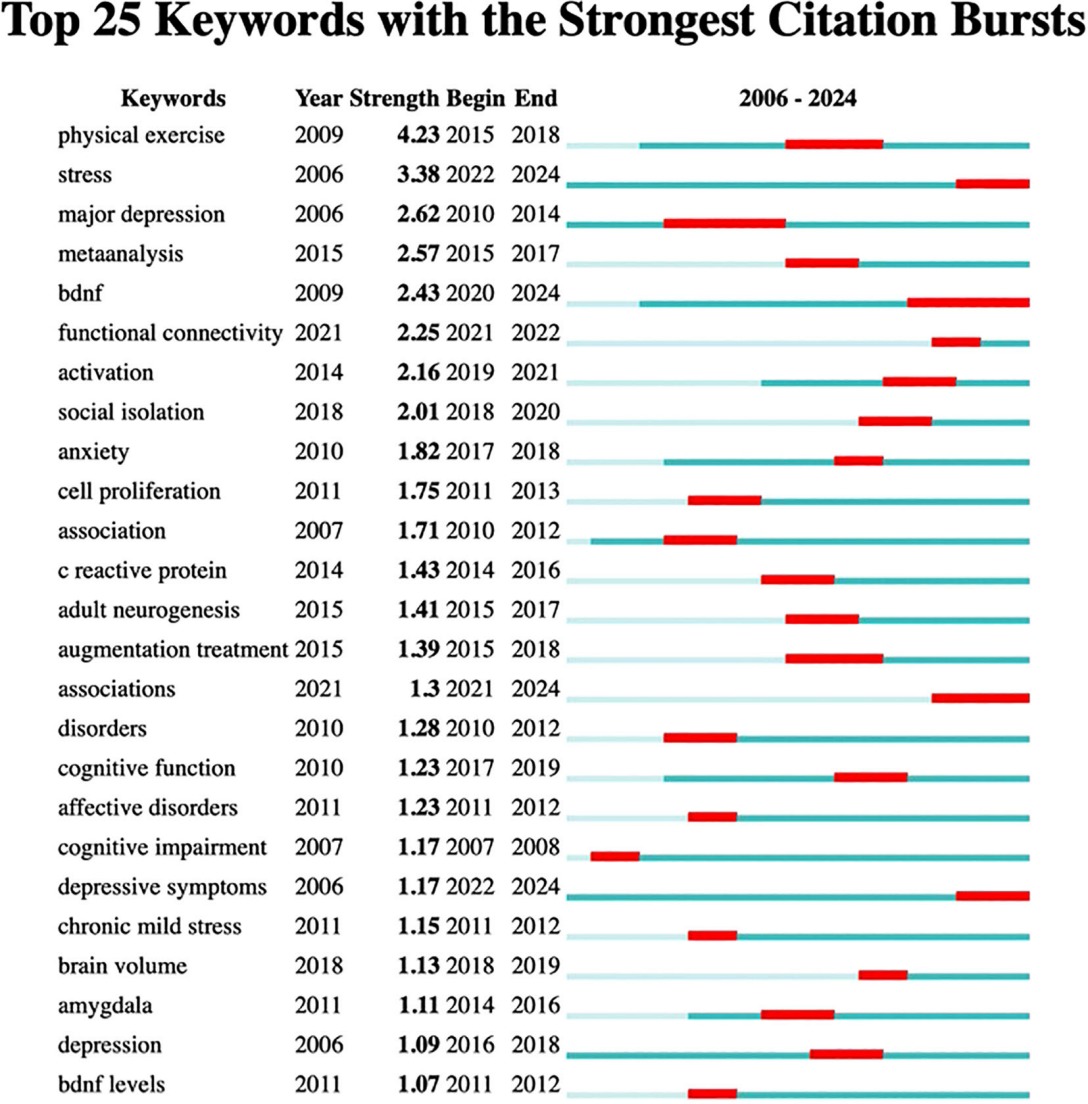
Top 25 Keywords with the strongest citation bursts.

Additionally, burst analysis depicted in **Figure 5** identified the six most vigorous keywords at the onset of their citation surges: “physical exercise” with an intensity of 4.23, “stress” at 3.38, “major depression” at 2.62, “BDNF” at 2.43, “meta-analysis” at 2.57, and “functional connectivity” at 2.25. Furthermore, the keyword “augmentation treatment” showed a low burst intensity of 1.39 during the period from 2015 to 2018, suggesting a slow advancement or ongoing debates in this area, particularly regarding drug combination therapies. The term “cell proliferation” experienced a brief burst from 2011 to 2013 with an intensity of 1.75, likely due to technical constraints, such as challenges in *in vivo* tracking, which led to diminished interest afterward. Similarly, “cognitive impairment” had an early burst from 2007 to 2008 with an intensity of 1.17 that was not sustained, likely reflecting a shift in focus toward emotional symptoms like depression and anxiety.

An examination of the evolution in the field, as illustrated by the emergence of keywords, reveals that from 2006 to 2014, the primary focus was on exploring fundamental mechanisms and interventions, with “major depression” highlighting biological processes such as the monoamine hypothesis and the development of pharmaceuticals. By around 2015, “physical exercise” shifted from being a peripheral topic to a recognized treatment option, supported by increasing clinical evidence. Between 2015 and 2020, the field began to embrace precision and interdisciplinary approaches. The years 2015 to 2017 were characterized by the standardization of research methodologies through meta-analyses and a push for evidence-based medicine regarding exercise interventions.

By 2022, advancements in neuroimaging techniques, such as fMRI, started to reveal brain network irregularities associated with “adult neurogenesis.” More recently, from 2021 to 2024, the focus has shifted towards “associations,” reflecting the rise of multi-omics, including genomic and epigenomic studies, as well as phenotypic investigations. Together, these emerging keywords illustrate the field’s progression from investigating pathological mechanisms to developing precise interventions. Future research should emphasize technology-driven, multimodal studies that explore brain networks and biomarkers, alongside clinical translational applications like personalized exercise prescriptions, while also addressing unresolved challenges such as the variability in the effectiveness of augmentation therapies.

### 3.3 Publication outputs and major journals

Our initial dataset included 337 references, but after applying the data filtering process described in the protocol, we excluded 50% of these references, resulting in a final dataset of 170 studies published between 2006 and 2024. These studies are all original articles, with an average of 6.36 authors per publication. Notably, the average number of co-authors increased from 5.8 during the period from 2006 to 2014 to 6.5 from 2015 to 2025. The earliest study in our dataset, conducted by Sarbadhikari (28), proposed and experimentally tested the hypothesis that moderate exercise could improve depressive symptoms by enhancing the activity of specific neurotransmitters.

**Figure 6:**
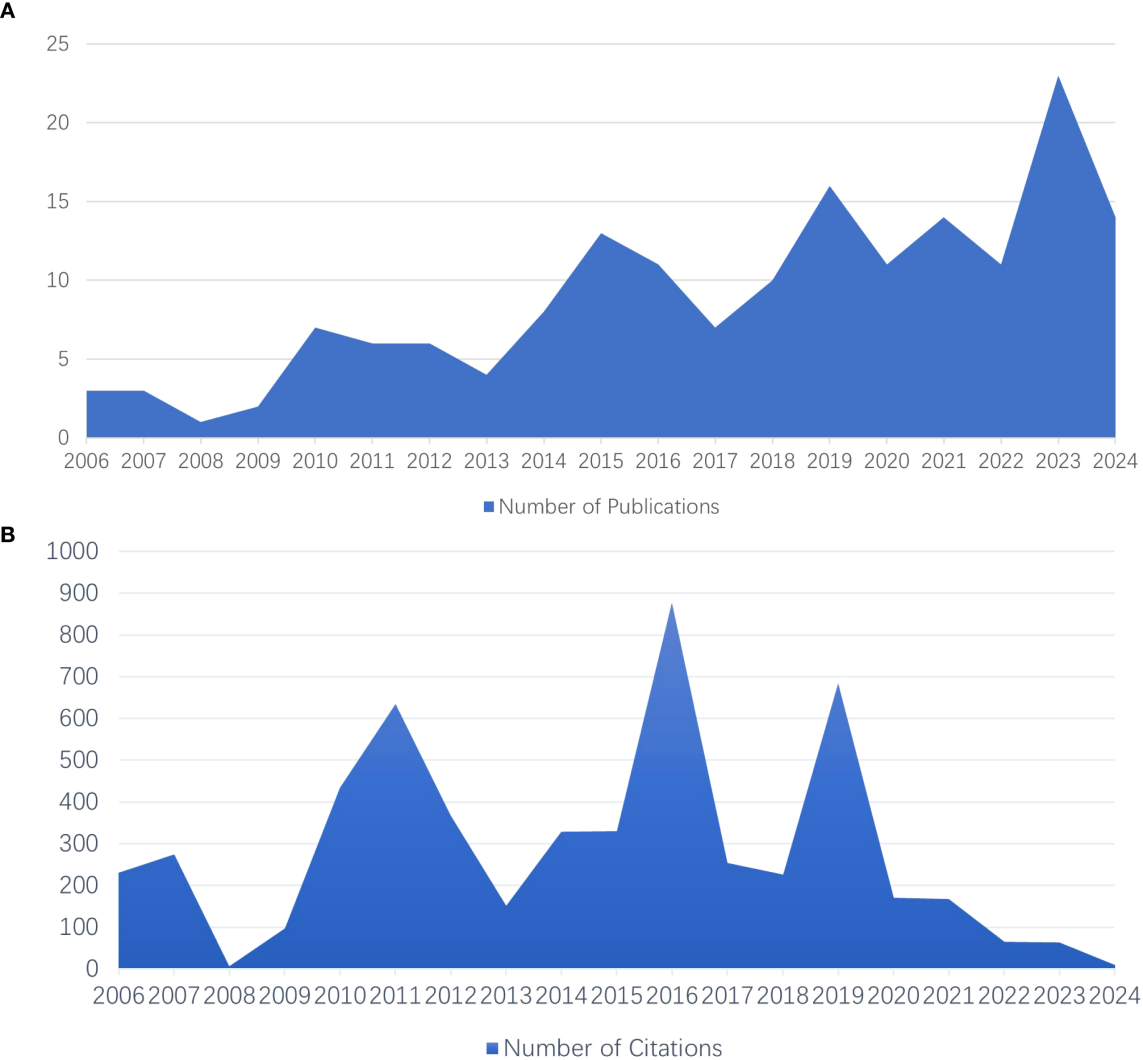
**(A, B)** Publication **(A)** and citation **(B)** performance in the field (2006-2024).

**Figures 6A, B** depict the evolution of publication outputs over time. In the initial period from 2006 to 2009, the annual number of publications was relatively low, ranging from 1 to 3 per year. However, starting in 2010, the number of publications began to fluctuate and increase, reaching peaks of 13 articles in 2015, 16 articles in 2019, and 23 articles in 2023. This sustained growth, particularly after 2015, is likely attributed to technological advancements, increased financial support, and enhanced academic collaboration. Interestingly, the number of citations did not always align with the volume of publications; for example, in 2016, there were high citation counts despite a lack of a corresponding increase in articles, indicating the significant impact of certain studies. Conversely, the high number of articles published in 2023 was associated with relatively low citation counts.

**Table 3** provides an analysis of the journals referenced in this study. The five most cited journals include Elife Sciences Publ Ltd, which leads with 498 citations, followed by Molecular Psychiatry with 348 citations, and both the Journal of Neuroscience and the Journal of Biological Chemistry, each with 316 citations. Additionally, Evidence-Based Complementary and Alternative Medicine has garnered 139 citations. In terms of publication volume, the Journal of Affective Disorders, established in 1979, and the Journal of Psychiatric Research, founded in 1966, are tied for the highest contribution, each accounting for 4.7% of the total articles, which amounts to 8 articles, with impact factors of 4.9 and 3.7, respectively.

**Table 3:** The top 10 most cited journals.

Top journals						
Journals with most articles (2006-2024)	Initial year	Impact factor(2024)	Total articles (%)	Total articles	Journals with most citations(2006-2024)	Total citations
JOURNAL OF AFFECTIVE DISORDERS	1979	4.9	4.7	8	eLIFE SCIENCES PUBL LTD	498
JOURNAL OF PSYCHIATRIC RESEARCH	1966	3.7	4.7	8	MOLECULAR PSYCHIATRY	348
FRONTIERS IN PSYCHIATRY	2010	5.4	4.1	7	JOURNAL OF NEUROSCIENCE	316
BEHAVIOURAL BRAIN RESEARCH	1993	2.6	2.9	5	JOURNAL OF BIOLOGICAL CHEMISTRY	72
PSYCHIATRY RESEARCH	1921	4.2	2.3	4	EVIDENCE-BASED COMPLEMENTARY AND ALTERNATIVE MEDICINE	139
TRANSLATIONAL PSYCHIATRY	2011	5.8	2.3	4	NEUROPHARMACOLOGY	135
BRAIN RESEARCH	1966	2.7	1.7	3	INTERNATIONAL JOURNAL OF NEUROPSYCHOPHARMACOLOGY	135
BRAIN SCIENCES	1878	2.8	1.7	3	NEUROREHABILITATION	126
NEUROSCIENCE	1981	2.9	1.7	3	NEUROSCIENCE	109
PHARMACOLOGY BIOCHEMISTRY AND BEHAVIOR	1973	3.3	1.7	3	JOURNAL OF AFFECTIVE DISORDERS	103

Moreover, Frontiers in Psychiatry, which began publishing in 2010, has quickly gained prominence, contributing 7 articles, or 4.1% of the total, with an impact factor of 3.2. Among the high-impact journals, Translational Psychiatry, founded in 2011 and boasting an impact factor of 5.8, has a relatively low publication rate, representing only 2.3% of the total publications with 4 articles, highlighting its commitment to high-quality research. In contrast, Pharmacology Biochemistry and Behavior, with an impact factor of 3.3, achieves a higher impact factor than some journals with greater publication volumes by focusing on specialized niche areas.

### 3.4 Collaborative network analysis between countries and institutions

**Figure 7:**
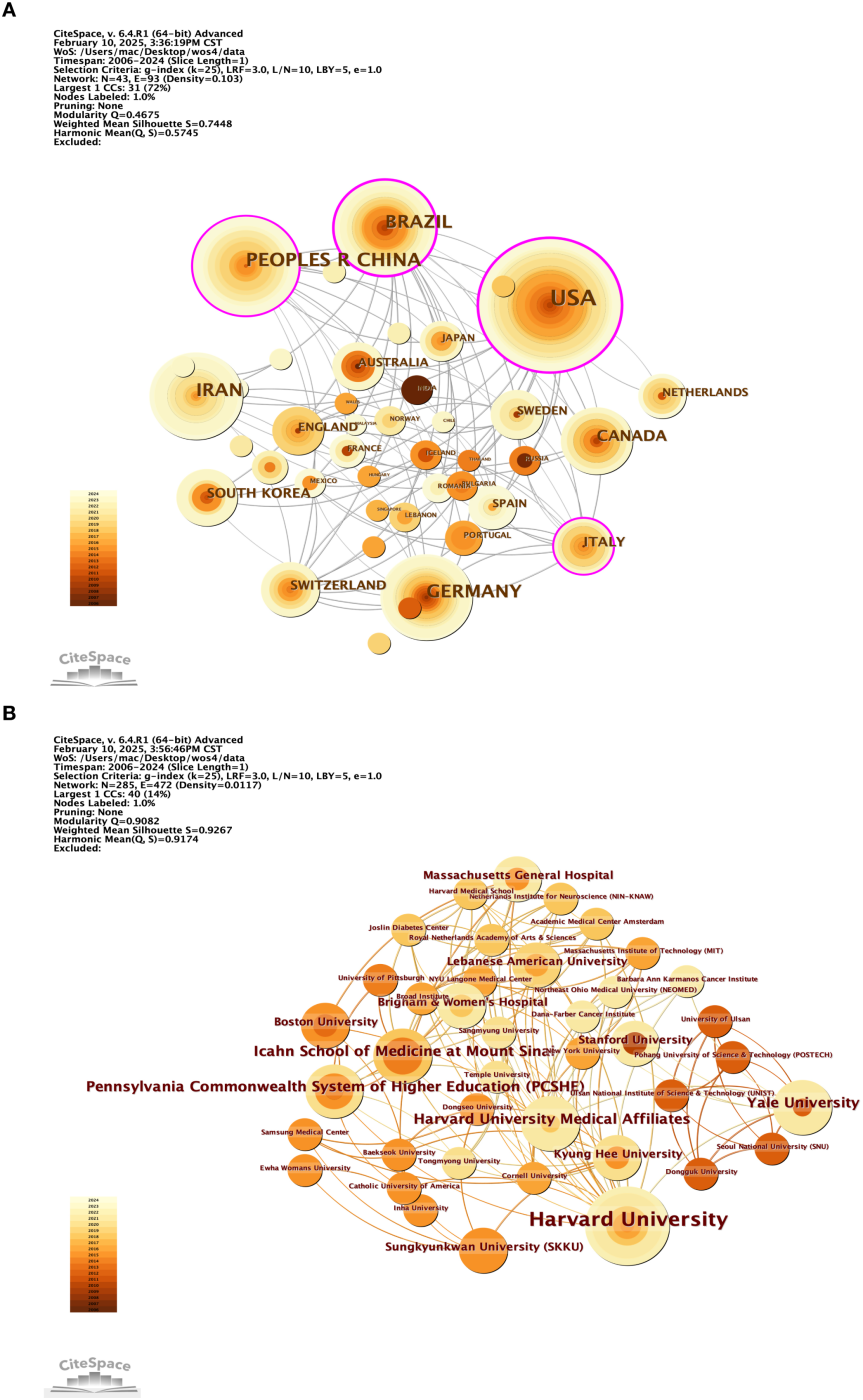
**(A, B)** 2006 to 2024 co-author country networks **(A)** and co-author institution networks **(B)** obtained with CiteSpace. **(A)** The visualisation of the network of co-author countries (based on the co-authors' countries) reveals the influence of each node. The network is organised by mediator centrality, with centrality scores normalised to the unit interval of [0,1]. Nodes with high mediated centrality are usually those connecting two or more large groups of nodes. Nodes with strong mediated centrality scores have a large impact on the network. High mediator centrality is indicated by the thickness of the purple trim. Nodes are restricted to the top 50 countries/regions. **(B)** Visualisation map of the network of co-authored institutions according to citation level. Annual citations for each institution are presented as citation tree rings. The most recent citation corresponds to the innermost ring. Nodes with purple coloured outermost rings are identified as hotspots. The colour of the link indicates the earliest time slice when the connection was first established.

As shown in **Figure 7A**, 43 countries have contributed to this field. Analysis of the frequency and centrality of these countries reveals that the United States (USA) has the highest frequency (45 occurrences) and centrality (0.31), underscoring its central role and high level of participation in the network. In contrast, although China and Germany show relatively high frequencies (22 and 19, respectively), their centrality values (0.14 and 0.05) are lower, suggesting that their contributions are less well connected within the overall network. Switzerland, with a frequency of 7 but a centrality of 0.09, plays a key role in connectivity, while Sweden, with 6 occurrences and a centrality of 0.04, has relatively weak participation and influence (see **Table 4**).

**Table 4:** Contributing countries by frequency and centrality.

Country\Region	Frequency	Country	Centrality
USA	45	USA	0.31
PEOPLES R CHINA	22	BRAZIL	0.21
BRAZIL	21	ITALY	0.15
GERMANY	19	PEOPLES R CHINA	0.14
IRAN	19	SWITZERLAND	0.09
CANADA	12	AUSTRALIA	0.07
ITALY	8	ENGLAND	0.07
SOUTH KOREA	8	GERMANY	0.05
SWITZERLAND	7	IRAN	0.05
SWEDEN	6	SWEDEN	0.04

**Figure 7B** indicates that a total of 446 organizations have contributed articles in this field. The institutions with the highest citation counts include Tehran University of Medical Sciences with 9 citations, Islamic Azad University with 8, Harvard University with 6, and both Humboldt University of Berlin and Karolinska Institutet with 5 citations each, as detailed in **Table 5**. An analysis of citation bursts over the past decade highlights that England exhibited the strongest citation burst (strength 2.03) from 2016 to 2019, followed by Iran (strength 1.55) from 2020 to 2024, Portugal (strength 1.57) from 2015 to 2016, and China (strength 1.22) from 2012 to 2021. These patterns suggest a consistent growth in research output, influenced by national initiatives like China’s “double first-class” program.

**Table 5:** Contributing institution by frequency and centrality.

Institutions	Frequency	Institutions	Centrality
Tehran University of Medical Sciences	9	Harvard University	0.02
Islamic Azad University	8	Yale University	0.01
Harvard University	6	Pennsylvania Commonwealth System of Higher Education (PCSHE)	0.01
Humboldt University of Berlin	5	Reykjavik University	0.01
Karolinska Institutet	5	Tehran University of Medical Sciences	0.01
Universidade Federal do Rio de Janeiro	4	Universidade Federal do Rio de Janeiro	0.01
Universidade Federal do Rio Grande do Sul	4	Islamic Azad University	0.01
University of Texas System	4	German Sport University Cologne	0.01
University of Texas Southwestern Medical Center Dallas	4	Kyung Hee University	0.01
Kermanshah University of Medical Sciences	4	Harvard University Medical Affiliates	0

**Figure 8:**
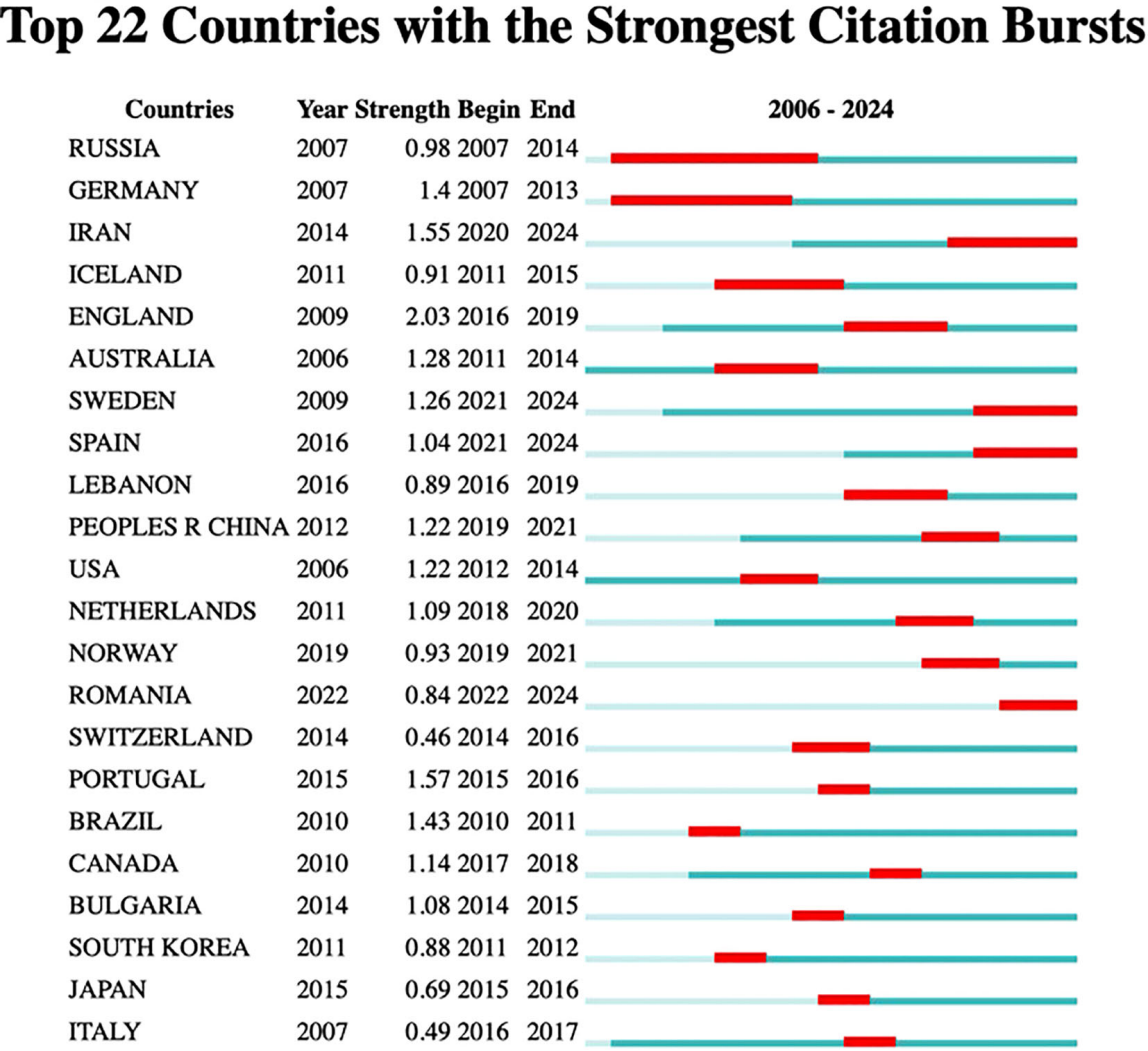
Top 22 Countries with the strongest citation bursts.

**Figure 9:**
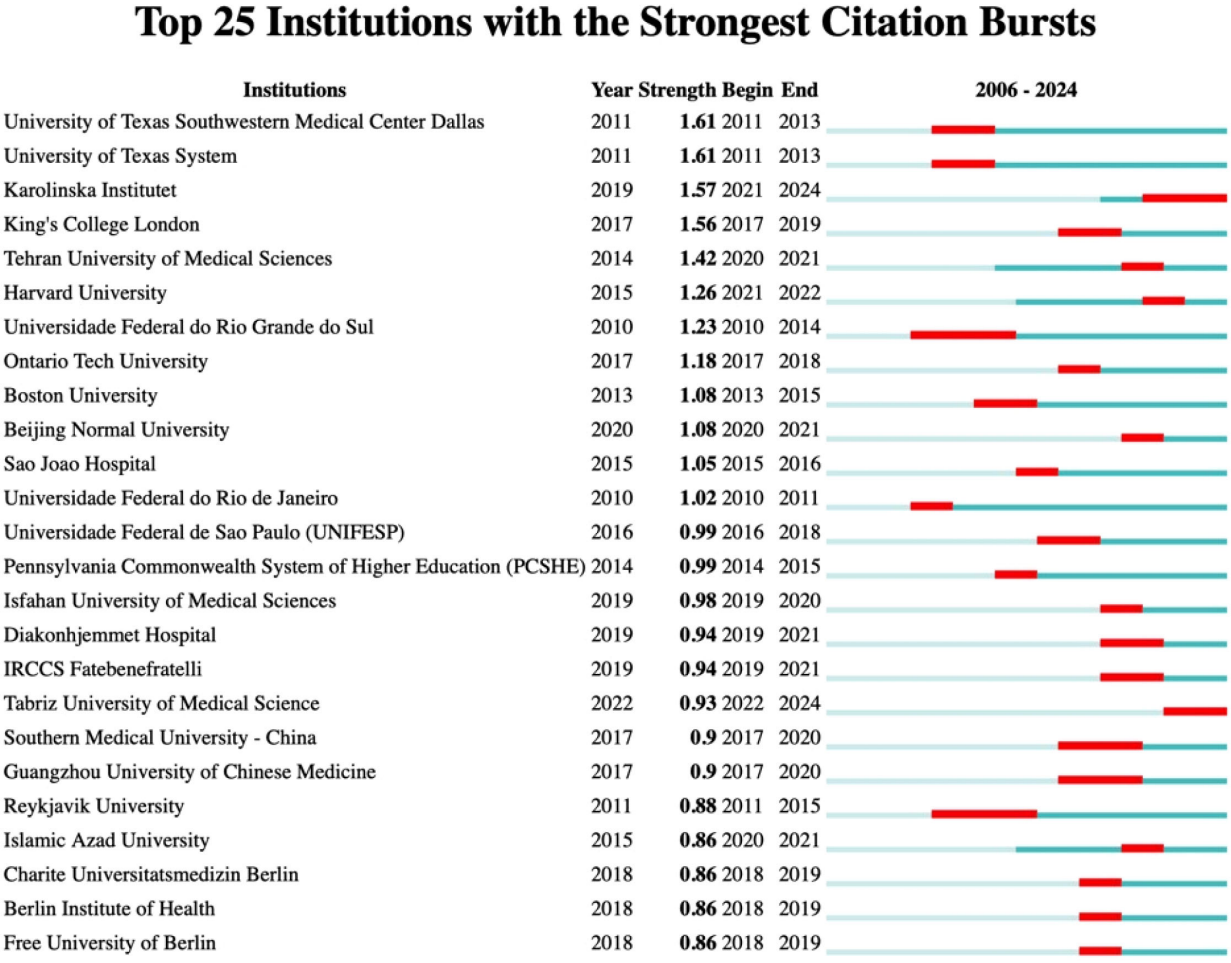
Top 25 Institution with the strongest citation bursts.

Among institutions, notable citation bursts were recorded for the University of Texas System (strength 1.61, 2011–2013) and the University of Texas at Urbana-Champaign (strength 2.03, 2016–2019), likely linked to significant advancements in cancer research and neuroscience, bolstered by NIH funding. Additionally, Karolinska Institutet (strength 1.57, 2021–2024) experienced a burst likely tied to research on COVID-19 vaccines and Nobel Prize-related studies, while King’s College London (strength 1.56, 2017–2019) has concentrated on psychiatry and neuroscience, particularly brain imaging studies on depression, supported by the UK Brain Programme. More comprehensive information can be found in **Figures 8** and **9**.

### 3.5 Collaboration network analysis

**Figure 10:**
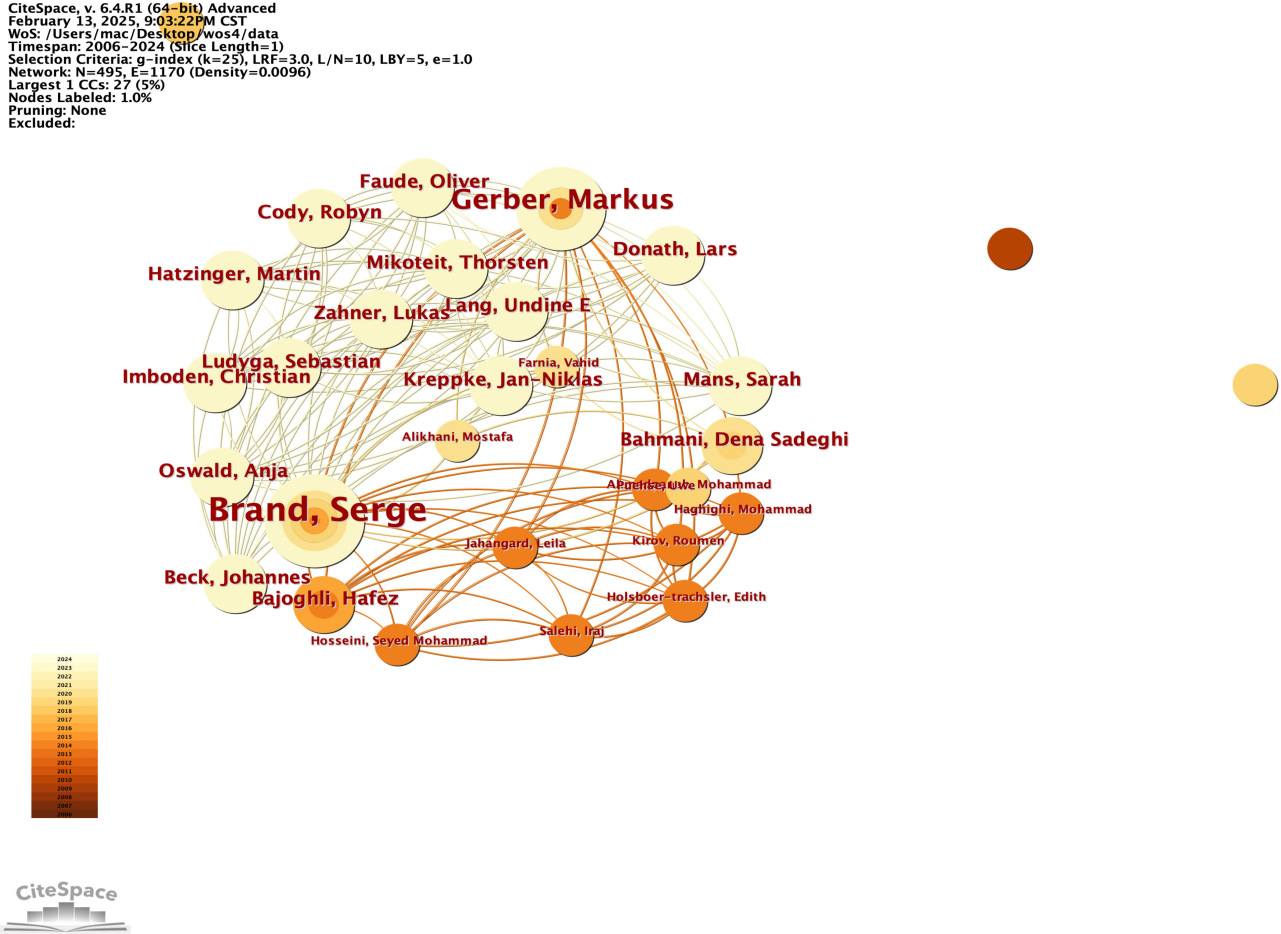
Authors' co-authorship networks from 2006 to 2024. The co-authorship network allows the visualisation of scientific collaborations between authors according to the frequency of co-authorship. A node represents an author. Links between each author represent collaborations (co-authorships). Both node and link colours indicate the year of the first collaboration (from dark 2006-light 2024). The size of the node is proportional to the number of co-authors of the author.

**Figure 11:**
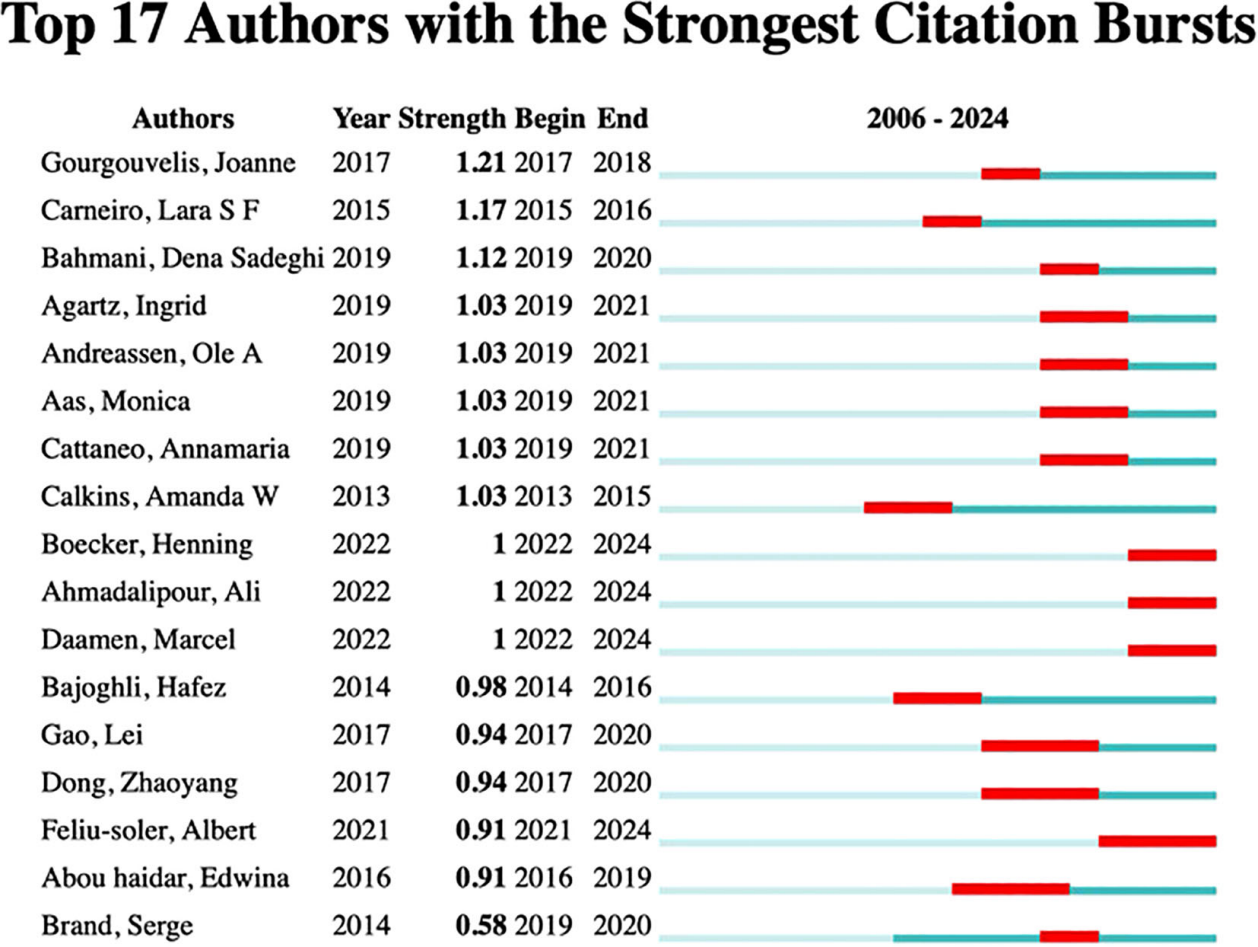
Top 17 Authors with the strongest citation bursts.

In this study, we developed a collaborative network of authors based on their co-authorship of papers that explore the neural mechanisms underlying physical exercise interventions in depression, as illustrated in **Figure 10**. We utilized an emergence detection technique to analyze these authors’ collaborative networks, their research activity, and their geographical distribution. The findings, presented in **Figure 11**, highlight prominent authors in this field, such as Carneiro, Lara S F (2015, intensity 117) and Gongpoweis, Joanne (2017, intensity 121), whose collaborative efforts were notably concentrated between 2015 and 2018. Furthermore, authors Brand, Serge from the University of Basel and Gerber, Markus showed significant publication outputs in 2014, with 6 and 5 papers respectively, as shown in **Table 6**. However, their citation burst intensity is relatively low, with Brand’s intensity at only 0.58. This suggests that their research may focus more on long-term academic contributions rather than generating immediate, short-term influence.

**Table 6:** Contribution authors (10or above).

Authors	Institutions	Publications	Year Begin
Brand, Serge	University of Basel	6	2014
Gerber, Markus	University of Basel	5	2014
Heinzel, Stephan	Free University of Berlin	4	2019
Stroehle, Andreas	Charite Universitatsmedizin Berlin	4	2019
Heissel, A.	University of Potsda	4	2019
Fydrich, T.	Humboldt University of Berlin	4	2019
Peeri, Maghsoud	Islamic Azad University, Central Tehran Branch	4	2023
Rethorst, Chad D.	Texas A&M University System	4	2023
Qi, Yingqiang	Chongqing Medical University	3	2023
Trivedi, Madhukar H.	University of Texas Southwestern Medical Center Dallas	3	2023

Since 2019, scholars such as Bahmani, Dona Sadeghi, and Agariz, Ingrid have demonstrated notable citation burst intensities, ranging from 1.03 to 1.12, while their collaborative networks have expanded across various institutions, including the Free University of Berlin, represented by researchers like Heinzel, and Islamic Azad University, showcasing a trend of diversified geographical cooperation. The cooperation pattern is predominantly European, with key institutions such as the University of Basel in Switzerland, the Free University of Berlin in Germany, and the University of the Basque Country in Spain acting as central hubs.

A significant aspect of this collaboration is the long-term partnership between Brand and Gerber, which has been ongoing since 2014, highlighting the strong academic connections between Switzerland and Germany. Additionally, institutions in the Berlin area, including Freie Universität Berlin and Humboldt Universität, have emerged as new cooperative nodes since 2019, likely influenced by Heinzel’s involvement. Furthermore, the participation of the Texas A&M University System in the USA and Chongqing Medical University in China illustrates a growing trend of cross-regional collaboration, emphasizing the increasing significance of cooperation among Europe, America, and Asia, and reflecting the internationalization of research efforts in this field.

## 4 Discussion

### 4.1 Summary of the main findings

This scientometric analysis systematically mapped the evolving neurobiological mechanisms linking exercise and depression across 2006-2024, revealing a clear three-phase progression: neurotransmitter-focused studies (2006–2014), pharmacological comparison trials (2015–2019), and precision intervention approaches (post-2020). The co-citation network analysis identified seven research clusters (Q=0.9299, S=0.9794) illustrating the field’s evolution from broad neurochemical investigations to molecular-level mechanisms, particularly emphasizing brain-derived neurotrophic factor (BDNF) signaling as a central pathway mediating exercise’s antidepressant effects (42). These findings provide clinicians with a roadmap for evidence-based exercise prescription, transitioning from generic recommendations to biomarker-guided, personalized interventions.

### 4.2 Identification of trends and clinical implications

#### 4.2.1 Evolution from neurotransmitter systems to molecular mechanisms

The comprehensive analysis of our co-citation reference network reveals critical translational pathways from bench science to clinical practice. The literature evolution from broad neurotransmitter systems (serotonergic, dopaminergic, GABAergic pathways) identified in early clusters to molecular-level investigations has direct implications for clinical biomarker monitoring (28). For example, the emerging emphasis on BDNF-mediated neuroplasticity and ketone body metabolism provides clinicians with potential biomarkers to monitor treatment efficacy objectively (49, 50). Keywords with high burstiness and centrality—“physical exercise,” “major depression,” “brain-derived neurotrophic factor,” and “cognitive function”—not only highlight influential research topics but also suggest specific clinical assessment targets for practitioners implementing exercise interventions. Our analysis of Cluster #5 studies suggests that monitoring peripheral BDNF levels at baseline and following 8-12 weeks of aerobic exercise could help clinicians identify biological responders versus non-responders, enabling timely intervention adjustments (43).

#### 4.2.2 Precision psychiatry applications

The identification of BDNF polymorphisms as moderators of exercise response in Cluster #3 has immediate implications for precision psychiatry. Clinicians could consider genetic screening for BDNF Val66Met polymorphisms to stratify patients who might require higher exercise “doses” or alternative exercise modalities to achieve comparable antidepressant effects (46). This genotype-guided approach represents a concrete application of personalized medicine principles in psychiatric care, moving beyond current one-size-fits-all exercise prescriptions.

#### 4.2.3 Exercise modality specificity and neurobiological mechanisms

A critical aspect requiring further clinical consideration is the type of physical exercise applied in studies exploring neurobiological mechanisms. While aerobic exercise is the most frequently reported modality, it encompasses diverse protocols including treadmill running, cycling, swimming, and dance-based interventions—each potentially activating distinct neurobiological pathways (51). Voluntary versus forced exercise has been shown to differentially modulate stress-response systems, with forced exercise potentially eliciting higher corticosterone responses than voluntary activity (52).

High-intensity interval training (HIIT) has been associated with different BDNF expression patterns compared to moderate continuous training, likely due to enhanced lactate production and subsequent neuroprotective signaling (53). These differences contribute to the heterogeneity of reported neurobiological outcomes and underscore the importance of specifying exercise types when developing personalized interventions.

#### 4.2.4 Emerging lactate-mediated mechanisms

An underexplored neurobiological mechanism relevant to exercise-induced antidepressant effects is the role of lactate. Traditionally viewed as a metabolic byproduct, lactate has recently been recognized as a key signaling molecule capable of crossing the blood-brain barrier and modulating neuroplasticity-related pathways (54). Preclinical studies demonstrate that peripheral lactate administration increases hippocampal BDNF expression and enhances long-term potentiation—both critical for mood regulation and cognitive resilience (52).

Furthermore, lactate appears to exert anti-inflammatory effects and modulate NMDA receptor activity, suggesting a multifaceted role in neural homeostasis (55). Although studies in our dataset did not consistently highlight lactate-related pathways, the growing evidence warrants its inclusion as a promising target for future research. Clinicians should consider that high-intensity exercise—which generates more lactate—might differentially influence antidepressant outcomes through this mechanism.

### 4.3 Clinical translation and implementation strategies

#### 4.3.1 Biomarker-guided treatment protocols

Our analysis of Cluster #11 research on ketone bodies suggests potential for integrating nutritional strategies with exercise protocols in clinical practice. The findings that β-hydroxybutyric acid enhances BDNF expression provide rationale for clinicians to consider combining ketogenic dietary approaches with exercise regimens, particularly for treatment-resistant cases (48). This integrated approach could be implemented in specialized depression clinics as an augmentation strategy when standard interventions prove insufficient.

The inflammatory hypothesis highlighted in recent literature offers clinicians additional biomarkers for monitoring treatment response. The decline in pro-inflammatory cytokines following exercise intervention could serve as accessible clinical markers, potentially more cost-effective than neuroimaging or complex genetic assays (47). Mental health practitioners could incorporate inflammatory marker panels (e.g., IL-6, TNF-α, CRP) into assessment protocols, particularly for patients with depression subtypes characterized by elevated inflammation.

#### 4.3.2 Evidence-based exercise prescription framework

The scientometric findings advocate moving beyond generic exercise recommendations toward structured, evidence-based prescriptions. Exercise modality specificity remains critical; differential effects of aerobic versus resistance exercise on BDNF expression indicate that aerobic interventions might be prioritized for patients with cognitive symptoms, whereas resistance training may benefit those with motivational deficits (44).

Equally important is exercise intensity calibration. Findings from Cluster #13 on symptom severity assessment suggest that moderate-intensity exercise (50-70% of maximum heart rate) sustained for 30-45 minutes optimizes neurobiological benefits while minimizing dropout rates—a key factor for clinical adherence (29). The emergence of dual-task paradigms provides clinicians with specific protocols to address cognitive impairments in geriatric depression, suggesting that integrating cognitive challenges with physical activity could enhance hippocampal neurogenesis more effectively than exercise alone.

### 4.4 Digital health technologies and future directions

#### 4.4.1 Real-time neurobiological monitoring

The scientometric analysis reveals an emerging trend at the intersection of exercise neurobiology and digital health technologies. The growing understanding of neurobiological mechanisms—particularly in Clusters #5 (Aerobic Exercise), #11 (Ketone Bodies), and #13 (Symptom Severity Assessment)—creates opportunities for technology-enabled, real-time optimization of exercise interventions.

The identification of BDNF as a key mediator suggests potential for wearable biosensor development that could monitor peripheral BDNF levels continuously. While traditional BDNF assessment requires laboratory blood analysis, emerging microfluidic and biosensor technologies show promise for detecting protein biomarkers in interstitial fluid, providing real-time feedback on neurobiological responses to exercise (56).

#### 4.4.2 Adaptive intervention platforms

Digital health platforms could leverage neurobiological data to create adaptive, personalized exercise prescriptions. For example, our analysis of dual-task paradigm research suggests that cognitive engagement during physical activity enhances neurobiological benefits. Digital platforms could integrate real-time cognitive assessment during exercise and adjust parameters to optimize cognitive-physical engagement (57).

Recent innovations in closed-loop neurofeedback systems offer models for how exercise interventions might be dynamically optimized. By integrating portable electroencephalography with mobile exercise platforms, changes in frontal alpha asymmetry—a neurophysiological marker of depression severity—could guide real-time adjustments to exercise intensity, duration, or modality.

### 4.5 Limitations and future research directions

One notable limitation of our study is the exclusive reliance on the Web of Science Core Collection, which may introduce citation bias by potentially excluding relevant studies from emerging journals or underrepresented regions. Additionally, the inherent data update lag may lead to omission of the most recent publications, affecting temporal analysis accuracy and identification of emerging trends.

To enhance representativeness and robustness of future bibliometric analyses, we propose integrating additional databases such as Scopus and PubMed. This multi-database approach would provide a more comprehensive literature pool, mitigate citation bias, and ensure analysis reflects current field developments (10).

Future research should prioritize multi-modal approaches integrating neuroimaging, metabolomics, and genomics to elucidate dose-response relationships and optimize exercise parameters. The development of smart digital intervention systems capable of providing real-time feedback and dynamically adjusting exercise protocols represents a tangible strategy for translating neurobiological insights into clinical practice. While our study provides foundational mapping of the field’s evolution, targeted clinical trials testing these precision intervention approaches are needed to validate their therapeutic efficacy.

## 5 Conclusion

Our scientometric analysis highlights the historical evolution, current trends, and emerging areas of research regarding the neurobiological mechanisms that explain how physical exercise affects depressive disorder. The findings not only validate the clinical efficacy of exercise as an adjunct treatment for depression but also underscore the need for multidisciplinary, personalized approaches. By using advanced bibliometric tools like CiteSpace, our study creates a solid framework to understand how neurobiological factors interact and to identify specific biomarkers that can guide personalized exercise prescriptions.

Additionally, our results indicate promising directions for future research, such as combining multi-modal neuroimaging with various omics data to clarify dose-response relationships and optimize exercise intensity, duration, and type. The development of smart digital intervention systems—capable of providing real-time feedback and dynamically adjusting exercise protocols—represents a tangible strategy for translating these scientific insights into clinical practice. While our study has limitations, such as relying on a single database and facing potential citation biases, it lays the groundwork for more targeted and effective therapeutic interventions that can be thoroughly assessed in future clinical trials.
