# Supplementary material for: Neurobiological mechanisms of the effect of exercise on depressive disorder: analysis using CiteSpace
Source: Front Psychiatry. 2025 Sep 2;16:1600286. doi: 10.3389/fpsyt.2025.1600286 (PMC12436359; doi:10.3389/fpsyt.2025.1600286)
Supplement: Supplementary file 1 [file Supplementaryfile1.docx]

**Full search syntax**

The full search terms were provided in the study protocol ((TS=(Exercises) OR TS=(Exercise, Physical) OR TS=(Exercises, Physical) OR TS=(Physical Exercise) OR TS=(Physical Exercise) OR TS=(Physical Exercises) OR TS=(Physical Exercises) OR TS=(Physical Activity) OR TS=(Physical Activity) OR TS=(Physical Activity) OR TS=(Physical Activity) OR TS= (Physical Activity) OR TS=(Activities, Physical) OR TS=(Activity, Physical) OR TS=(Physical Activities) OR TS=(Physical Activities) OR TS=(Exercise, Aerobic) OR TS=(Aerobic Exercise) OR TS=(Aerobic Exercises) OR TS=(Exercises, Aerobic) OR TS=(Exercise, Isometric) OR TS=(Exercises, Isometric) OR TS=(Isometric) Exercises) OR TS=(Isometric Exercise) OR TS=(Acute Exercise) OR TS=(Acute Exercises) OR TS=(Exercise, Acute) OR TS=(Exercises, Acute) OR TS=(Exercises, Acute) OR TS=(Exercise Training) OR TS=(Exercise Trainings) ) And ((TS=(Depressive Disorders) OR TS=(Disorder, Depressive) OR TS=(Disorders, Depressive) OR TS=(Disorders, Depressive) OR TS=(Neurosis, Depressive) OR TS=(Depressive Neuroses) OR TS=(Depressive Neurosis) OR TS=(Neuroses, Depressive) OR TS=(Depression, Endogenous) OR TS=(Depressions, Endogenous) OR TS=(Endogenous Depression) OR TS=(Endogenous Depressions) OR TS=(Melancholia) OR TS=(Melancholias) OR TS=(Unipolar Depression) OR TS=(Depression, Unipolar) OR TS=(Depressions, Unipolar) OR TS=(Unipolar Depressions) OR TS=(Depressive Syndrome) OR TS=(Depressive Syndromes) OR TS=(Syndrome, Depressive) OR TS=(Syndromes, Depressive) OR TS=(Depression, Neurotic) OR TS=(Depressions, Neurotic) OR TS=(Depressions, Neurotic) OR TS=(Neurotic Depression) OR TS=(Neurotic Depressions)) AND ((TS=(neurobiology) OR TS=(brain function) OR TS=(neurophysiology) OR TS=(neuroscience) OR TS=(biological mechanisms) OR TS=(neurotransmitters) OR TS=(brain plasticity) OR TS=(brain-derived neurotrophic factor) OR TS=(amygdala) OR TS = (prefrontal cortex)).
